# Decreased serum obestatin consequent upon *TRIB3* Q84R polymorphism exacerbates carotid atherosclerosis in subjects with metabolic syndrome

**DOI:** 10.1186/1758-5996-4-52

**Published:** 2012-12-17

**Authors:** Ai-dong Cui, Ning-ning Gai, Xiu-hua Zhang, Ke-zhi Jia, Yan-li Yang, Ze-jun Song

**Affiliations:** 1 Yantai Municipal Laiyang Central Hospital, Yantai, 265200, People’s Republic of China; 2 Department of Cardiology, No 111, Changshan Road, Laiyang, Shandong, People’s Republic of China

**Keywords:** Obestatin, *TRIB3*, Metabolic syndrome, Carotid atherosclerosis

## Abstract

**Background:**

Functional *TRIB3* Q84R polymorphism has been associated with insulin resistance. Obestatin, improving insulin resistance, exerts obscure effects on metabolic syndrome (MetS) and carotid atherosclerosis. Aims to investigate whether the prevalent *TRIB3* Q84R polymorphism has profound implications for alterations of serum obestatin and what effect obestatin exerts on carotid atherosclerosis.

**Methods:**

A total of 518 unrelated Chinese subjects consisted of control (n = 258) and MetS (n = 260) groups. Clinical and biochemical characteristics were collected. The level of serum obestatin was measured. Genotype the functional *TRIB3* Q84R polymorphism. All subjects underwent ultrasonography to determine carotid intima-media thickness (IMT).

**Results:**

Serum obestatin was significantly decreased in MetS as compared with the control group (*P* = 0.042). Among the MetS group participants possessing RR84 genotype had significantly lower levels of serum obestatin than those with QQ84 or QR84 genotypes (*P* = 0.008, *P* = 0.043) with similar significant difference among the control group. Factorial analyses showed statistically significant interactions between MetS and RR84 genotype (*P* = 0.009 for interaction for obestatin). On correlation analysis, obestatin correlated negatively with homeostasis model assessment insulin resistance (*r* = -0.163, *P* = 0.010) and IMT (*r* = -0.256, *P* = 0.011). On partial analyses, obestatin negatively correlated with IMT(*r* = -0.259, *P* = 0.024) after controlling for the confounding factors.

**Conclusion:**

MetS individuals with *TRIB3* RR84 genotype demonstrated further decreased serum obestatin. Decreased serum obestatin might in part exacerbate insulin resistance and carotid atherosclerosis.

## Introduction

The existence of metabolic syndrome (MetS) implies a shift from a pathophysiology concept based on metabolic abnormalities resulting from an insulin-resistant state to an epidemiological construct based on abdominal obesity and crude correlates of the features of insulin resistance. Excessive circulating adipocytokines might provide pathways linking abdominal obesity to insulin resistance, which has led several groups to try to identify secreted products derived specifically from this depot. Obestatin [1], reducing food intake, body weight gain, gastric emptying, and jejunal motility, has been demonstrated to be involved in insulin resistance and metabolic dysfunctions. The functional profiles of obestatin reported in the past studies would suggest a regulator of adipocyte metabolism by promoting adipogenesis in an autocrine/paracrine manner [2,3], which pointing to a putative role in the pathogenesis of metabolic syndrome. However, at present the pathophysiological role of obestatin in MetS remains unknown.

Studies addressing the molecular mechanisms have revealed that obestatin regulates adipocyte function and protects against diet-induced insulin resistance and inflammation [3]. As previously reported, obestatin is decreased in obese subjects [4]. However, whether decreased obestatin ameliorates or deteriorates insulin resistance remains obscure. Furthermore, what effects obestatin exerts upon carotid atherosclerosis remains unknown.

*TRIB3* (a mammalian tribbles homolog, also known as TRIB3/NIPK, gene ID 57761), has been reported by most studies [5,6], although not all studies [7], implicated in the regulation of insulin signal transduction by binding to and inhibiting Akt phosphorylation and to play a role in insulin resistance [5,6]. Furthermore, it has been demonstrated that the *TRIB3* gene, especially the R84 variant, could help identify individuals at risk for insulin resistance and carotid atherosclerosis risk by determining carotid intima-media thickness (IMT) [8]. As mentioned above, obestatin might go down with MetS, the key culprit of which is insulin resistance. Therefore, obestatin might be involved in MetS. However, whether the prevalent *TRIB3* missense Q84R polymorphism has profound implications for alterations of serum obestatin and what effect obestatin exerts remain to be established.

In the present study, we aimed to investigate the role of obestatin in MetS and carotid atherosclerosis under the background of *TRIB3* Q84R polymorphism by case-control study.

## Materials and methods

A total of 518 unrelated homogenous Chinese subjects between the ages of 30 and 77 years were recruited from the Yantai Municipal Laiyang Central Hospital of Shandong: 260 subjects [159 men; mean age 55 ± 9 years] with MetS, defined according to the 2005 criteria of the International Diabetes Federation; 258 subjects (controls) [155 men; mean age 54 ± 8 years] without cardiovascular diseases or elevated fasting glucose (fasting plasma glucose ≥ 5.6 mmol/L) and with waist circumference less than 90 cm for men and 80 cm for women, defining central obesity, conducted from September 20, 2008 to June 2, 2011. Written informed consent was obtained from all subjects before enrollment in the study, and procedures were approved by the ethics committees of Yantai Municipal Laiyang Central Hospital of Shandong and followed the Helsinki Declaration criteria.

Subjects were advised to refrain from strenuous physical activity, smoking, eating and drinking for at least 12 h before the screening visit. Screening included the completion of standardized questionnaires collecting personal information and the collection of data on age, sex, personal medical history, and history of coronary heart disease, hypertension, dyslipidemia, and diabetes. Systolic blood pressure (SBP) and diastolic blood pressure (DBP) were measured twice in the right arm then averaged for subjects who had been resting for at least 10 min in a comfortable position. Waist circumference was measured at the midpoint between the iliac crest and the lower rib margin and hip circumference around the maximum circumference of the buttocks posteriorly and the symphysis pubis anteriorly.

### Laboratory measurements

After blood samples were taken, plasma preserved with EDTA and serum were separated immediately by centrifugation at 2500 *g* for 10 min. Glucose measurements involved the glucose oxidase method. Total cholesterol and total triglyceride levels were determined by enzymatic methods. HDL-C was measured in the supernatant after precipitation of apo B-containing lipoproteins with use of phosphotungstic acid and magnesium chloride. LDL-C level was calculated by use of the Friedewald formula. LDL-C was not calculated for individuals with triglyceride level >4.5 mmol/L. Serum concentration of insulin was determined by use of a radioimmunometric assay kit (Dongya Ltd, Beijing). Intra- and interassay coefficients of variation for insulin were 10% and 15%, respectively; no cross-reactivity was observed between insulin and proinsulin. Insulin resistance was assessed from glucose and insulin concentrations by the use of the homeostasis model assessment (HOMA) equation, which equals to fasting blood glucose multiplied by fasting insulin then divided by 22.5 [9]. Details about the anthropometric and biochemical parameters refer to Table 1.

**Table 1:** Demographic characteristics of the studied population

**Characteristics**	**Control group**	**MetS group**	***P***
	**(n = 258)**	**(n = 260)**	
Sex (male/female)	155/103	159/101	0.802
Age (years)	54 ± 8	55 ± 9	0.182
WC (cm)	82.08 ± 7.69	97.42 ± 9.44	<0.001
SBP (mmHg)	114.90 ± 10.85	151.29 ± 22.82	<0.001
DBP (mmHg)	75.03 ± 7.34	93.47 ± 13.70	<0.001
TG (mmol/L)	1.02 ± 0.40	2.32 ± 1.30	<0.001
HDL-C (mmol/L)	1.56 ± 0.36	1.22 ± 0.29	<0.001
FBG (mmol/L)	4.83 ± 0.60	6.61 ± 2.48	<0.001
Insulin (μU/mL)	10.63 ± 4.73	20.78 ± 11.20	<0.001
Log(Obestatin)	109.65 ± 14.79	95.50 ± 13.80	<0.001

Data are means ± SD for normally-distributed variables.

Abbreviations: MetS, metabolic syndrome; WC, Waist circumference; SBP, systolic blood pressure; DBP, diastolic blood pressure; TG, triglycerides; HDL-C, high-density lipoprotein cholesterol; FBG, fasting blood glucose.

### Serum obestatin assay

Serum obestatin was measured by a commercially available RIA kit (Phoenix Pharmaceuticals Inc., Mountain View, CA, USA) following the manufacturer’s instruction. Samples were measured in duplicate in a single experiment. The intra- and interassay coefficients of variance of this kit are < 5% and < 14%, respectively.

### Genotyping

Genomic DNA was prepared from blood leukocytes by established methods. Genotyping of the *TRIB3* R84 variant involved the RFLP-PCR method with the primers 5^′^- GGC CAC CAA GCA GTC TCAC -3^′^ (forward) and 5^′^- CGC CCA TGA TCC CTA AGT TC -3^′^ (reverse). PCR conditions and protocols were as described by Prudente et al. [10]. Screening was performed by adding 1 unit of MspI restriction enzyme (Takara Biotechnology(Dalian) Co.,Ltd) into 15 μL of PCR product. After 2-h incubation at 37°C, products were loaded onto 1.5% agarose gel and visualized by staining with ethidium bromide. For genotyping, positive and negative controls (known sequences after sequencing) were used, and the rate of genotyping success for the SNP was >99%. (Additional file 1: Figure S1).

### Echocardiography

All subjects underwent echocardiographic examination with a commercially available ultrasound machine (Vivid 7 dimension; General Electric Medical Systems, Horten, Norway) equipped with a 2.5 MHz variable-frequency scanner as reported [8].

## Statistical analysis

The Kolmogorov-Smirnov test was used to test for normal distribution. Normally distributed data are presented as means ± SD, and non-normally distributed data are presented as medians (quartile range). Non-normally distributed variables were given a log-transformation if necessary. Continuous variables were compared between groups by unpaired Student’s *t* test or Mann-Whitney *U* test and among groups by one-way ANOVA with post-hoc LSD *t* test when appropriate. The chi-square test was used to analyze the associations between categorical variables and for Hardy-Weinberg equilibrium. The correlation between 2 variables was assessed by Pearson or Spearman correlation coefficient analysis. After controlling for covariates, bivariate correlations underwent partial correlation analysis. Next, multivariable models were constructed to study the effects of obestatin on heart, with other study variables taken into account as covariates. A *P* value <0.05 was considered significant whether obtained from a one-tailed or two-tailed test of significance. Analyses involved SPSS v. 13.0 (SPSS Inc., Chicago, IL).

## Results

### Baseline demographic features

Genotyping was successful in 518 subjects. The *TRIB3* Q84R polymorphism genotypes were in Hardy-Weinberg equilibrium (Table 2). Demographic characteristics of the study population are in Table 1. Serum obestatin was significantly decreased in MetS as compared with the control group. Demographic characteristics among different genotype subgroups are provided in Additional file 2: Tables S1 and S2.

**Table 2:** Genotype and allele frequency of TRIB3 Q84R polymorphism in subjects with and without MetS

	**QQ84**	**QR84**	**RR84**	**Q84**	**R84**
Control group	168	70	20	406	110
MetS group	155	79	26	389	131

Abbreviations: MetS, metabolic syndrome.

### Effects of TRIB3 polymorphism on serum obestatin

Among the control group, the obestatin concentrations of QQ84, QR84 and RR84 were 151.36 ± 11.75, 102.33 ± 14.45 and 95.50 ± 17.39, respectively; significant differences were observed between groups. Among the MetS group, participants possessing RR84 genotype had significantly lower levels of serum obestatin than those with QQ84 or QR84 genotypes (Table 3). Furthermore, MetS patients possessing RR84 genotype had even lower levels of serum obestatin than controls with RR84 genotype (70.79 ± 19.50 vs 95.50 ± 17.39, *P* = 0.0001). The similar results in QQ84 genotypes.

**Table 3:** **Comparison of levels of serum obestatin by *TRIB3 *Q84R genotype**

	**QQ84**	**QR84**	**RR84**	***P *for ANOVA**
Control group (n = 258)	151.36 ± 11.75	102.33 ± 14.45^**^	95.50 ± 17.39^**^†	<0.001
MetS group (n = 260)	112.20 ± 14.79	100.01 ± 16.98^**^	70.79 ± 19.50^**^†	<0.001

Data are means ± SD. ^**^P < 0.01 vs QQ84 genotype, † *P* < 0.05 vs QR84 genotype.

Abbreviations are as in Table 1.

### Association of decreased obestatin with carotid atherosclerosis in metabolic syndrome patients

What effect decreased obestatin exerts on carotid artery remains an open question. Correlation analysis showed that obestatin had a negative correlation with TC and LDL (*r* = -0.202, *P* = 0.018; *r* = -0.205, *P* = 0.016; respectively). Obestatin was negatively correlated with carotid IMT (*r* = -0.256, *P* = 0.011).

To get further insight into the association of serum obestatin with carotid IMT in subjects with MetS, partial analyses were performed. On partial analyses, obestatin was negatively correlated with carotid IMT (*r* = -0.259, *P* = 0.024) after controlling for sex, age, SBP, DBP, waist circumference (WC), triglycerides, HDL-C, LDL-C, fasting blood glucose (FBG), insulin and smoking.

Furthermore, the factors with statistically significant associations with carotid IMT in the multivariable model were age (β = 0.319, *P* = 0.001) and obestatin (β = -0.294, *P* = 0.003, Table 4).

**Table 4:** Multivariable models of the relationship of obestatin with carotid atherosclerosis, adjusted for clinical characteristics and cardiovascular risk factors in subjects with MetS

**Variables**	***β***	***P***
carotid IMT		
Age	0.319	0.001
Obestatin	-0.294	0.003

Abbreviations are as in Table 1.

## Discussion

To the best of our knowledge, this is the first study that aims to elucidate the interplay between MetS, *TRIB3* polymorphism and obestatin. In the present study *TRIB3* RR84 genotype with MetS saw further decreased obestatin. Furthermore, lowered obestatin might play an independent role in carotid atherosclerosis.

### TRIB3 polymorphism and obestatin

*TRIB3* is implicated in the regulation of insulin signal transduction [5] by binding to serine-threonine kinase PKB/Akt and blocking its activation. *TRIB3*, thus, is speculated to contribute to insulin resistance. Of note, a progressive reduction of insulin-induced Ser^473^ Akt phosphorylation was observed from untransfected control to Q84- and R84-expressing HepG2 cells with no changes of Akt protein expression [10]. However, obestatin enhanced glucose uptake in either the absence or presence of insulin, promoted GLUT4 translocation, and increased Akt phosphorylation, therefore reducing insulin resistance [3]. As mentioned above, since TRIB3 induces insulin resistance which would be reversed in part by obestatin, an important question has been whether the *TRIB3* polymorphism would determine the level of serum obestatin.

Our results indicated that *TRIB3* RR84 variant interacts significantly with MetS on the level of serum obestatin. As reported previously [4], obese subjects had significantly lower levels of obestatin. However, no genotype of the *TRIB3* altered the level of serum obestatin among the control group. Further factorial analyses showed that *TRIB3* RR84 variant will decrease the level of serum obestatin only in subjects with MetS.

In addition, it is important to acknowledge that only homozygous *TRIB3* RR84 variant could decrease the level of serum obestatin in MetS individuals while heterozygous *TRIB3* QR84 or homozygous *TRIB3* QQ84 variants have no such, which suggested that here Q84 allele is dominant with R84 allele recessive.

### Obestatin and carotid artery in metabolic syndrome

Most intriguing of all, our results showed that serum obestatin correlates inversely with IMT independent of the other potential affecting factors, which suggested that obestatin might have a bad effect on carotid atherosclerosis, otherwise obestatin may be a biomarker.

## Limitation

Despite intriguing findings, the present results require confirmation by a prospective population study, since the MetS cohort was not all made up of newly diagnosed subjects. However, this study provides new insights into the potential associations of decreased obestatin with *TRIB3* Q84R polymorphism, MetS and carotid atherosclerosis. In fact, without the foundation provided by the present study, the ensuing prospective population study could not be implemented.

## Conclusion

In conclusion, our results confirmed that serum obestatin decreases with MetS, but further decreases due to *TRIB3* RR84 genotype. Incremental serum obestatin might in part protect against carotid atherosclerosis.

## Competing interests

The authors declare that they have no competing interests.

## Authors’ contributions

ADC carried out the molecular genetic studies, participated in the sequence alignment and drafted the manuscript. NNG carried out the laboratory measurements, serum obestatin assay. XHZ participated in the Genotyping and Echocardiography. KZJ and ZJS participated in the design of the study and performed the statistical analysis. YLY and ADC conceived of the study, and participated in its design and coordination and helped to draft the manuscript. All authors read and approved the final manuscript.

## Supplementary Material

Additional file 1: Figure S1 Electrophoresis results on incubation with MspI restriction enzyme. Click here for file

Additional file 2: Table 1 Demographic characteristics of Control group by TRIB3 Q84R genotype. **Table 2.** Demographic characteristics of MetS group by TRIB3 Q84R genotype. Click here for file
