# Supplementary figures and images for: Decreased serum obestatin consequent upon TRIB3 Q84R polymorphism exacerbates carotid atherosclerosis in subjects with metabolic syndrome
Source: Diabetol Metab Syndr. 2012 Dec 17;4:52. doi: 10.1186/1758-5996-4-52 (PMC3573955; doi:10.1186/1758-5996-4-52)

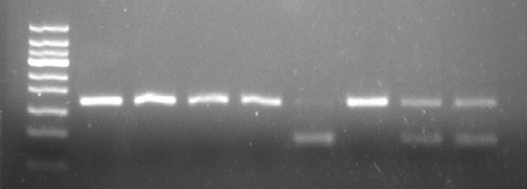

Supplement: Additional file 1: Figure S1 — Electrophoresis results on incubation with MspI restriction enzyme. [file 1758-5996-4-52-S1.tiff]
