# Supplementary material for: Decreased serum obestatin consequent upon TRIB3 Q84R polymorphism exacerbates carotid atherosclerosis in subjects with metabolic syndrome
Source: Diabetol Metab Syndr. 2012 Dec 17;4:52. doi: 10.1186/1758-5996-4-52 (PMC3573955; doi:10.1186/1758-5996-4-52)
Supplement: Additional file 2: Table 1 — Demographic characteristics of Control group by TRIB3 Q84R genotype. Table 2. Demographic characteristics of MetS group by TRIB3 Q84R genotype. [file 1758-5996-4-52-S2.doc]

Table 1 Demographic characteristics of Control group by *TRIB3* Q84R genotype

| Characteristics | QQ  （n=168） | QR  （n=70） | RR  （n=20） | *P* |
| --- | --- | --- | --- | --- |
| Sex (male/female) | 100/68 | 45/25 | 10/10\ | 0.510 |
| Age (years) | 54±9 | 53±8 | 54±9 | 0.717 |
| WC (cm) | 81.12±8.59 | 82.42±9.04 | 84.12±7.44 | 0.247 |
| SBP (mmHg) | 112.75±9.95 | 115.09±12.12 | 115.58±10.82 | 0.211 |
| DBP (mmHg) | 74.83±10.34 | 75.47±9.70 | 75.32±9.60 | 0.482 |
| TG (mmol/L) | 0.96±0.30 | 1.01±0.72 | 1.02±0.68 | 0.708 |
| HDL-C (mmol/L) | 1.61±0.35 | 1.42±0.39 | 1.38±0.27 | 0.0001 |
| FBG (mmol/L) | 4.78±0.59 | 4.82±0.48 | 4.84±0.39 | 0.817 |
| Insulin (μU/mL) | 9.98±3.78 | 10.85±10.10 | 10.78±9.20 | 0.610 |
| Log(Obestatin) | 151.36±11.75 | 102.33±14.45 | 95.50±17.39 | <0.001 |

Table 2 Demographic characteristics of MetS group by *TRIB3* Q84R genotype

| Characteristics | QQ  （n=155） | QR  （n=79） | RR  （n=26） | *P* |
| --- | --- | --- | --- | --- |
| Sex (male/female) | 98/57 | 50/29 | 11/15 | 0.120 |
| Age (years) | 54±9 | 55±7 | 55±10 | 0.652 |
| WC (cm) | 88.90±7.16 | 96.92±10.47 | 99.82±8.15 | <0.001 |
| SBP (mmHg) | 141.89±9.58 | 153.19±11.72 | 158.79±12.92 | <0.001 |
| DBP (mmHg) | 85.93±8.39 | 95.77±11.69 | 97.87±9.70 | <0.001 |
| TG (mmol/L) | 1.92±0.38 | 2.42±1.19 | 2.65±1.90 | <0.001 |
| HDL-C (mmol/L) | 1.36±0.28 | 1.20±0.31 | 1.01±0.11 | <0.001 |
| FBG (mmol/L) | 5.83±0.64 | 6.72±2.33 | 7.21±2.31 | <0.001 |
| Insulin (μU/mL) | 19.63±5.73 | 21.88±9.20 | 21.52±11.34 | 0.080 |
| Log(Obestatin) | 112.20±14.79 | 100.01±16.98 | 70.79±19.50 | <0.001 |
